# Mechanisms of Long Non-Coding RNAs in the Assembly and Plasticity of Neural Circuitry

**DOI:** 10.3389/fncir.2017.00076

**Published:** 2017-10-23

**Authors:** Andi Wang, Junbao Wang, Ying Liu, Yan Zhou

**Affiliations:** ^1^Hubei Key Laboratory of Cell Homeostasis, College of Life Sciences, Wuhan University, Wuhan, China; ^2^Medical Research Institute, Wuhan University, Wuhan, China

**Keywords:** long non-coding RNA, neural circuit, cell fates, synaptogenesis, synaptic plasticity, CRISPR-Cas9

## Abstract

The mechanisms underlying development processes and functional dynamics of neural circuits are far from understood. Long non-coding RNAs (lncRNAs) have emerged as essential players in defining identities of neural cells, and in modulating neural activities. In this review, we summarized latest advances concerning roles and mechanisms of lncRNAs in assembly, maintenance and plasticity of neural circuitry, as well as lncRNAs' implications in neurological disorders. We also discussed technical advances and challenges in studying functions and mechanisms of lncRNAs in neural circuitry. Finally, we proposed that lncRNA studies would advance our understanding on how neural circuits develop and function in physiology and disease conditions.

## Introduction

The human brain confers on us the abilities of perceptions, thoughts, emotions, actions, and memories. Over many years, the neuroscientists have strived to understand the molecular, cellular, circuit and behavior-level mechanisms that underlie these processes. Around a century ago, Santiago Ramon *y* Cajal proposed the neuron doctrine postulating that the relationship between nerve cells was not continuous, but contiguous. Cajal, in his *Theory of Dynamic Polarization*, described how information, in the form of electrical signals, travels within individual neurons, from their dendrites to their cell bodies and finally to their axons. We now know cognition, emotion, memory, and action are generated by circuits and networks of thousands to millions of interconnected neural cells, mostly neurons. Neural circuits are both anatomical and functional entities, composed of a series of interconnected neurons and glial cells with diverse properties and functions. However, it remains largely elusive how specific types of neural cells assembles the neural circuits in different brain regions and how specific neural circuits perform their signal processing functions during cognitive processes and behaviors. This requires detailed knowledge on the construction of neural circuits at the single-cell resolution and on the spatiotemporal pattern of neuronal activity (Poo et al., 2016). The United States BRAIN (Brain Research through Advancing Innovative Neurotechnologies) initiative was launched in 2013, which was intent to “*accelerate the development and application of innovative technologies to construct a dynamic picture of brain function that integrates neuronal and circuit activity over time and space*,” To achieve this goal “*requires an integrated view of its component cell types, their local and long range synaptic connections, their electrical and chemical activity over time, and the functional consequences of that activity at the levels of circuits, the brain, and behavior*” (NIH, 2014).

The process of neuronal specification, migration, and circuit formation is enormously complex in time and space during development, with multiple levels of regulation. Deciphering this process requires both a high-throughput neuronal subclass identification and an integrative approach that considers dynamic, multilayered transcriptional regulation (Molyneaux et al., 2015). However, current regulatory models are limited to a number of regulators, mostly transcription factors, which account for a limited subset of key nodes within a broader regulatory network that is believed to be far more complex (Greig et al., 2013). The transient expression, flexible structures, and dynamic localization of RNA molecules enable fine-tuning genome arrangement, scaffolding and transcription functions, thus precisely regulating gene expression in a time and site-specific manner. Recent work indeed points to the critical role of long non-coding RNAs (lncRNAs) in transcriptional and post-transcriptional regulation of gene expression, the formation of complexes with epigenetic regulatory machinery, and chromosomal architecture organization (Rinn and Chang, 2012; Quinn and Chang, 2016). Therefore, lncRNAs participate in numerous physiological and pathological processes including maintenance of pluripotency, lineage specification, organogenesis, tumorigenesis, and metabolism (Wang et al., 2011; Ramos et al., 2013; Li and Chang, 2014; Yang et al., 2014; Wu et al., 2015). Although recent reviews have covered many aspects of lncRNAs in the assembly and plasticity of neural circuits, this field is fast-growing with new evidence reinforcing the notion that lncRNAs are pivotal in cell fate determination and in modulating neural activity (Ng et al., 2013a; Aprea and Calegari, 2015; Briggs et al., 2015). In this review, we highlighted roles and mechanisms of lncRNAs in assembly, maintenance, plasticity and abnormality of neural circuitry. Given the *cis-* and *trans-* regulatory mechanisms by lncRNAs and/or their embedding DNA elements, along with far more uncharacterized lncRNAs than protein-coding genes, strategies and technologies in studying lncRNAs were also discussed. Finally, we speculate that findings in lncRNA studies would deepen our understanding on neural circuitry composition and functional dynamics in physiology and disease conditions.

## LncRNAs are abundant in brain and display spatiotemporal specificity

Current data from the ENCODE consortium suggest that up to 75% of the human genome may be transcribed (Djebali et al., 2012), but only about 1–2% of the human genome seems to encode protein (Birney et al., 2007; Church et al., 2009). Thus, most transcripts are non-protein-coding RNA (ncRNA) transcripts (Chodroff et al., 2010). LncRNAs are usually defined as non-protein coding transcripts longer than 200 nucleotides (nt) to exclude small regulatory RNAs such as short interfering RNAs (siRNAs), microRNAs (miRNAs), small nucleolar RNAs (snoRNAs), Piwi-interacting RNAs (piRNAs), and other short RNAs. Occasionally, functional short peptides can be derived from lncRNAs (Matsumoto et al., 2017). Until now, the NONCODE database have annotated 101,700 lncRNA genes in the human genome (Zhao et al., 2016). Remarkably, 40% of lncRNAs are expressed predominantly in the brain (Derrien et al., 2012). While many lncRNA genes overlap protein-coding genes in sense or antisense directions, others resides in genomic regions previously termed “gene deserts,” between protein-coding genes (intergenic) (Carninci et al., 2005; Cheng et al., 2005; Katayama et al., 2005; Kapranov et al., 2007a,b; Qureshi et al., 2010). In a recent study, using FANTOM5 (Functional Annotation of Mammalian cDNA) cap analysis of gene expression (CAGE) data, Hon et al. generated an atlas of nearly 30,000 human lncRNA genes with typical 5′ ends and expression profiles across 1,829 human primary cell types and tissues. Interestingly, most intergenic lncRNAs (lincRNAs) originate from enhancers rather than from promoters. Incorporating genetic and expression data implicates around 20,000 potentially functional lncRNAs in multiple diseases and in transcriptional regulation (Hon et al., 2017).

Some lncRNAs have distinct molecular biogenesis features compared to protein-coding transcripts. Using native elongating transcript sequencing (mNET-seq), Schlackow et al. found human lincRNAs and protein-coding pre-mRNAs are transcribed by different Pol II phospho-CTD (the C-terminal domain) isoforms. LincRNAs are rarely spliced, mainly non-polyadenylated, and are stabilized in the nucleoplasm (Schlackow et al., 2017). LncRNA conservation includes four dimensions: the sequence, structure, function, and expression from syntenic genome loci (Diederichs, 2014). In fact, lncRNA exons are significantly more conserved than neutrally evolving sequences, albeit at lower levels than protein-coding genes (Derrien et al., 2012). Interestingly, lncRNA promoters are more conserved than their exons, and nearly as conserved as promoters of protein-coding gene (Guttman et al., 2009).

LncRNAs are generally expressed at lower levels than protein-coding transcripts, and exhibit more cell- and tissue-specific expression patterns. Moreover, lncRNA expression is vigorously regulated during neural development (Mercer et al., 2010; Belgard et al., 2011; Aprea et al., 2013; Molyneaux et al., 2015) and upon neural activity (Lipovich et al., 2012; Barry et al., 2014), suggesting their specific functional roles. Analyzing *in situ* hybridization data from ABA (the Allen Brain Atlas), numerous lncRNAs are found to be expressed in the adult mouse brain and most of them were present in specific neuroanatomical structures or cell types such as particular cortical regions or the hippocampus (Mercer et al., 2008). Similarly to the expression of fate-determining protein-coding genes, these region-specific and dynamic expression patterns of lncRNAs could be orchestrated by *cis*-regulatory elements (enhancers), chromatin status, and cell-type-specific or activity-dependent transcription factors (TFs) (Ramos et al., 2013).

## LncRNAs control neural cell fates via *cis*- and *trans*- regulatory mechanisms

Production of neurons and glial cells during neural development is an intricate but highly stereotyped process that necessitates accurate spatiotemporal controlling of neural stem/progenitor cells (NSPCs) self-renewal and differentiation (Zhou, 2012). The mature mammalian neocortex, for example, is a multi-layered structure and the layers-specific projection neurons are generated sequentially by cortical neural stem/precursor cells (NSPCs) lying in the ventricular zone/subventricular zone (VZ/SVZ) over developmental time. Intriguingly, cortical NPCs acquire restrictions in fate potential progressively over developmental time, which are largely cell-intrinsic (Mcconnell, 1995; Desai and Mcconnell, 2000; Shen et al., 2006; Gaspard et al., 2008). In contrast, cortical interneurons, which usually make local and inhibitory connections, are produced mostly from precursors in the ventral telencephalon and cortical hem and undergo tangential migration into the cortex (Anderson et al., 2002; Wonders and Anderson, 2006). Notably, most neurons are not directly derived from bipolar radial glial neural precursor cells (RGCs) but are from multipolar intermediate progenitor cells, which are direct progenies of RGCs and may undergo a few rounds of replication prior to differentiation. This so-called indirect cortical neurogenesis is more prevalent in primates than in rodents (Qian et al., 2000; Franco and Muller, 2013; Greig et al., 2013). Cortical neurogenesis is followed by gliogenesis, which occurs mostly after birth in mice.

### *Cis*-acting lncRNAs

**Figure 1:**
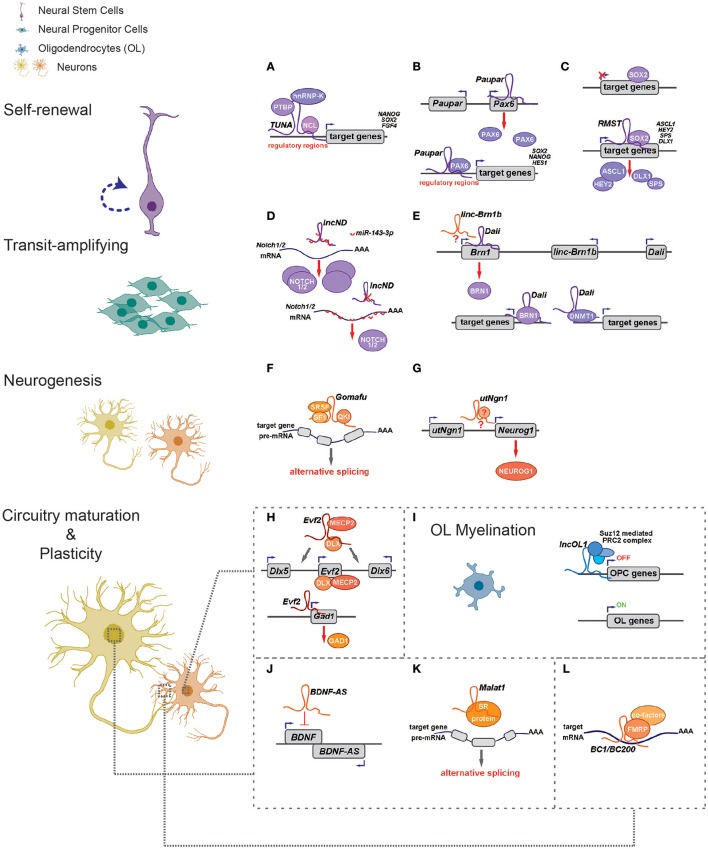
LncRNAs regulate different aspects of neural circuitry assembly and function (left) *via cis*- and *trans*- mechanisms (right). **(A–G)** LncRNAs can control neurogenesis of neural stem/progenitor cells *via* regulating expressions of proximal genes **(B,E,G)** and/or distal genes by associating with fate-determining transcription factors **(A–C,E)**, acting as competing endogenous RNAs **(D)** and regulating alternative splicing **(F)**. **(H)** *Evf2* controls expression of *Dlx5*/*Dlx6 in cis* and *Gad1 in trans* to regulate GABAergic interneuron specification. **(I)** *LncOL1* interacts with SUZ12 to repress a gene program that maintains oligodendrocyte progenitor state, thereby promoting OL myelination. **(J–L)** LncRNAs regulate neurite outgrowth and synaptogenesis *via cis*-regulation **(J)**, alternative splicing **(K)**, and translational control **(L)**.

Recent studies unveil that lncRNAs have essential regulatory roles balancing NSPC self-renewal and differentiation (Figures 1A–I; Table 1). Initially, researchers were interested in lncRNAs transcribed from loci overlapping with or adjacent to transcription factor (TF) genes known to be essential for NSPC fate choices. The rationale is based on the facts that lncRNAs can regulate transcription locally (*in cis*) (Wang et al., 2008; Guil and Esteller, 2012; Dimitrova et al., 2014; Engreitz et al., 2016; Luo et al., 2016). One of such examples, *utNgn1*, is a noncoding RNA transcribed from an enhancer region (3.8–7.8 kb upstream of the transcription start site) of mouse *Neurogenin1* (*Neurog1*), a key TF that promotes neuronal fate specification (Figure 1G). Expression pattern of *utNgn1* is highly correlated with that of *Neurog1* mRNA. Moreover, *utNgn1* is required for the expression of *Neurog1* during neuronal differentiation of cortical NPCs (Onoguchi et al., 2012).

**Table 1:** Examples of lncRNA activities in neural circuitry assembly and function.

**Process**	**LncRNA**	**Cell/tissue distribution**	**Biological function/phenotypes**	**Molecular mechanisms: *Cis* and/or *trans* regulation**	**Protein partner**
Neural stem cell proliferation	*Pnky*	Mouse and human NSPCs	Regulates neuronal differentiation of embryonic and adult NSPCs	*Trans*: *Pnky* and PTBP1 regulate the expression and alternative splicing of an overlapping set of transcripts to promote neurogenesis	PTBP1
	*LncND*	Radial glia NSPCs	Promotes expansion of radial glial NSPCs	*Trans*: *LncND* compete with the 3′UTR of *NOTCH1* or *NOTCH2* for the binding of miR-143-3p to promote Notch signal pathway	/
	*Paupar*	Neuro-2a neuroblastoma cells^*^	Knockdown of *Paupar* induces neural differentiation of Neuro-2a neuroblastoma cells	*Cis*: *Paupar* regulates *Pax6* expression locally. *Trans*: *Paupar* also associates with PAX6 protein and localizes at promoters of *SOX2, NANOG*, and *HES1*	PAX6
	*utNgn1*	Mouse NSPCs	Promotes neuronal differentiation	*Cis*: *utNgn1* is transcribed from an enhancer region of *Neurog1* and positively regulates its expression	/
Neuronal differentiation	*RMST*	Midbrain dopaminergic neuronal precursor cells	Promotes neuronal differentiation	*Trans*: *RMST* interacts with SOX2 to regulate neurogenic genes including *ASCL1* and *DLX1*	SOX2, hnRNPA2/B1
	*Tuna*	Mouse ESCs; mouse and zebrafish CNS	Regulates pluripotency and neural differentiation of ESCs	*Trans*: *TUNA* formed a complex with three pluripotency related RNA-binding proteins, PTBP1, hnRNP-K, and NCL	PTBP1, HNRNP-K, NCL
	*Linc-Brn1b*	Developing mouse neocortex: SVZ and upper cortical layers	Specifies cortical NSPC fate and regulate area patterning and layer formation of mouse neocortex	*Cis*: Deletion of the *linc-Brn1b* locus leads to significant decrease in *Brn1* expression	/
	*NBAT-1*	SH-SY5Y cells^*^	Loss of *NBAT-1* increases cellular proliferation and invasion	*Trans*: *NBAT-1* interacts with EZH2 to suppress expression of *SOX9, OSMR*, and *VCAN*	EZH2
	*Miat/Gomafu*	NSPCs, neurons	Controls retinal development. Dysregulated in schizophrenia	*Trans*: *Gomafu*/*Miat* regulates splicing of neuronal genes, including *DISC1, ERRB4*, and *WNT7B*, probably via association with splicing factors SF1, SRSF1, and QKI	QKI, SRSF1, SF1
	*Dali*	Neuro-2a neuroblastoma cells^*^	Depletion of *Dali* in Neuro-2a neuroblastoma cell inhibits its neuronal differentiation induced by retinoic acid	*Cis*: *Dali* maintains *Brn1* expression. *Trans*: *Dali* interacts with the DNMT1 to regulates DNA methylation status of CpG island-associated promoters; interacts with BRN1 to regulate expression of neural differentiation genes	BRN1, DNMT1
Neurites outgrowth and synaptogenesis	*Kcna2-as*	DRG sensory neurons	Upregulation of *Kcna2-as* decreased *Kcna2* mRNA, reduced total voltage-gated potassium current, increased excitability in DRG neurons, ultimately leading to neuropathic pain symptoms	*Cis*: *Kcna2-as* silences *Kcna2* expression	/
	*BC1/BC200*	Dendrites and somata of neurons	Regulates synaptic excitability	*Trans*: *BC1* controls protein translation in synaptodendritic microdomains	FMRP, eIF4A, PABP
	*BDNF-AS*	DRG sensory neurons	Depletion of *BDNF-AS* promotes neuronal outgrowth and adult neurogenesis	*Cis*: *BDNF-AS* negatively regulates *BDNF* expression by recruiting EZH2, a PRC2 core component	EZH2
	*Malat1*	Differentiated projection neurons	Promotes dendrite maturation and synaptogenesis in cultured hippocampal neurons	*Trans*: *Malat1* associates with SR family splicing factors to controls expression of synaptic molecules including *Nlgn1* and *SynCAM1*	SF2/ASF, SC35
Interneurons	*Evf2*	Postmitotic interneurons	Ensures proper formation of GABA-dependent neuronal circuitry	*Cis* and *trans*: *Evf2* associates with DLX1/2 and MECP2 at the regulatory elements in the *Dlx5/6* intergenic region to control *Dlx5, Dlx6* and *Gad1* expression	DLX1, DLX2, BRG1, MECP2
Glial cells	*Six3OS*	Retinal progenitor cells	Regulates retinal cell specification, neuron and oligodendrocyte differentiation	*Trans*: *Six3OS* binds to EZH2 and EYA family members to regulate expression of SIX3 target genes	EZH2, EYA1, EYA3, EYA4
	*LncOL1*	Mature oligodendrocytes (OLs)	Promotes oligodendrocyte myelination	*Trans*: *LncOL1* interacts with SUZ12 to suppress a gene program that maintains OL progenitor state, thereby promoting OL myelination.	SUZ12

* *Research subject; ESCs, embryonic stem cells; NSPCs, neural stem/progenitor cells; V-SVZ, ventricular-subventricular zone; DRG, dorsal root ganglion*.

Interestingly, many such lncRNAs can simultaneously target distal genes by associating with *cis*-elements in the genome, TFs and epigenetic modifiers. LncRNA *Evf2* (also known as *Dlx6os1* or *Dlx6as*) is transcribed from the ultra-conserved intergenic region between the *Dlx5* and *Dlx6* genes, encoding two members of the DLX homeodomain-containing protein family essential for interneuron development (Feng et al., 2006). Disruption of mouse *Evf2* transcription results in decreased numbers of GABAergic interneurons in early postnatal hippocampus and dentate gyrus, and reduced synaptic inhibition in CA1 layer of the adult hippocampus (Bond et al., 2009). Mechanistically, *Evf2* controls the expression of interneuron lineage genes, including *Gad1, Dlx5*, and *Dlx6*, by both *cis*- and *trans*-acting mechanisms. *Evf2* guides methyl-CpG-binding protein MECP2 and the transcription factor DLX to regulatory elements in the *Dlx5/6* intergenic region, thus regulating *Dlx5/6* expression by modulating the opposing interactions between DLX and MECP2, and by modulating *Dlx5/6* ultra-conserved enhancer site-specific methylation (Figure 1H; Berghoff et al., 2013). *Paupar* (*Pax6 Upstream Antisense RNA*) is a single-exon lncRNA transcribed from 8.5 kb upstream of the *Pax6* gene in mouse, which is evolutionarily conserved in term of genomic organization and sequence. Knockdown of *Paupar* induces neural differentiation of Neuro-2a neuroblastoma cells. *Paupar* acts locally to regulate *Pax6* expression in a transcript-dependent manner. Interestingly, *Paupar* also functions *in trans* to control distal neural gene expression on a large scale. *Paupar* transcript physically associates with PAX6 protein and localizes at promoters of *Sox2, Nanog*, and *Hes1* to regulate cell-cycle progression and differentiation of Neuro-2a cells (Figure 1B; Vance et al., 2014).

*Linc-Brn1b* is transcribed from a 6.8 kb genomic locus about 10 kb downstream of the *Brn1* (*Pou3f3*), a well-studied TF gene involved in cortical development. *Linc-Brn1b*'s expression is restricted in germinal zones (VZ/SVZ) of the early developing brain but becomes prominent in the cortical plate neurons in late cortical neurogenesis, indicating its role in regulating neuronal differentiation. Deletion of the *linc-Brn1b* locus leads to significant decrease in *Brn1* expression. Moreover, *linc-Brn1b*-null cerebral cortices displayed an expression signature indicative of decreased cellular proliferation and increased neuronal differentiation. Consistently, the embryonic cortices of *linc-Brn1b*-null mice showed decreased numbers of intermediate progenitors and upper layer (II-IV) projection neurons, accompanied by an expansion of deep layer neurons. Furthermore, *linc-Brn1b-*null mice exhibit reduced barrel size and number in the somatosensory cortex. All these suggest *linc-Brn1b* specifies cortical NPC fate and regulate area patterning and layer formation. However, it's elusive the cortical defects found in *linc-Brn1b-*null mice are due to depletion of *linc-Brn1b* transcript or the deletion of its embedding *cis* element (Sauvageau et al., 2013). *Dali*, another lncRNA transcribed downstream of *Brn1* (*Pou3f3*) locus, also controls neuronal differentiation partly *via* its positive regulation of *Brn1* expression. Depletion of *Dali* in Neuro-2a neuroblastoma cell inhibits its neuronal differentiation induced by retinoic acid. Intriguingly, similar to *Paupar, Dali* also interacts directly with the protein product of its neighboring genes, BRN1, to regulate expression of a large set of neural differentiation genes *in trans*. Moreover, *Dali* associates with DNMT1, a DNA methyltransferase, to regulate DNA methylation status of promoter CpG islands (Figure 1E; Chalei et al., 2014).

### *Trans*-acting lncRNAs

Recently, several lncRNAs are reported to mainly function *in trans* by directing TFs or chromatin modifiers to important loci, thus regulating expression of distal genes that are essential for cell fate specifications. *Pnky* is a conserved nuclear lncRNA predominantly expressed in NSPCs of both the embryonic and postnatal brain. Depletion of *Pnky* promotes neuronal lineage specification and augments the population of transit-amplifying cell, leading to increased neurogenesis. RNA pull-down assay identified PTBP1, an RNA-splicing factor that potentiates neural development and neuronal reprogramming (Keppetipola et al., 2012; Xue et al., 2013), as the binding partner of *Pnky*. In NSPCs, *Pnky* and PTBP1 promote neurogenesis by regulating the expression and alternative splicing of an overlapping set of transcripts (Ramos et al., 2015).

Rhabdomyosarcoma 2-associated transcript (*RMST*), a lncRNA with prominent expression in midbrain dopaminergic neuronal precursors, is required for neuronal differentiation of human ESCs (Uhde et al., 2010; Ng et al., 2012). *RMST* is negatively regulated by the transcription factor REST and upregulated during neuronal differentiation. *RMST* interacts with SOX2 to co-activate a large pool of neurogenic genes such as *ASCL1, NEUGOG2, HEY2* and *DLX1* to promote neuronal differentiation (Figure 1C; Ng et al., 2013b). *TUNA* (Tcl1 Upstream Neuron-Associated lincRNA, or megamind), a highly conserved lincRNA that show specific expression in developing CNS of zebrafish and mice, was required for pluripotency maintenance and proper neural differentiation of mouse embryonic stem cells. *TUNA* formed a complex with three pluripotency related RNA-binding proteins (RBPs), hnRNP-K, PTBP1, and NCL, at the promoters of *Nanog, Sox2*, and *Fgf4* to regulate gene expression. Furthermore, disruption of *TUNA* expression in zebrafish caused impaired locomotor function (Figure 1A; Lin et al., 2014).

### Competitive endogenous RNAs

Some lncRNAs contains multiple complementary sites for microRNAs (miRNAs). These competitive endogenous RNAs (ceRNAs) act as molecular sponges for miRNAs through their miRNA binding sites (also known as miRNA response elements, MRE), thereby de-repressing all target genes of the respective miRNA family (Cesana et al., 2011; Salmena et al., 2011; Tay et al., 2014). Human *lncND* (neurodevelopment) contains 16 MREs for miR-143-3p and is highly expressed in the progenitor zone (VZ/SVZ) in developing human neocortex where it co-localizes with NSPC markers such as PAX6. *LncND* positively regulates the expression of *NOTCH1* and *NOTCH2*, two receptors genes essential for NSPC self-renewal, by competing the binding of miR-143-3p to NOTCH1/2′s 3′ untranslated region (UTR). Depletion of *lncND* induced neuronal differentiation of neuroblastoma cells, an effect reminiscent of miR-143-3p overexpression (Figure 1D; Rani et al., 2016). Circular RNAs or transcripts of pseudogenes might also behave as ceRNAs (see next section for more details).

### LncRNAs in myelination

LncRNAs also have roles in glial cell fate determination. Oligodendrocytes provide support and insulation to axons in the central nervous system of some vertebrates by creating the myelin sheath. Diseases that result in injury to the oligodendrocytes include demyelinating diseases such as multiple sclerosis and various leukodystrophies. Many lncRNAs are dynamically expressed during oligodendrocyte (OL) lineage specification, neuronal-glial fate switches, and OL lineage determination such as myelination (Mercer et al., 2010; Dong et al., 2015; He et al., 2017). In an integrative analysis using transcriptomic and epigenetic data, Dong et al. characterized lncRNAs that are differentially expressed in the process of oligodendrocyte precursor cell (OPC) differentiation from mouse neural stem cells (NSCs) and that are potential regulators of oligodendrogenesis (Dong et al., 2015). Co-expression network analyses associates distinct oligodendrocyte-expressing lncRNA clusters with protein-coding genes and predict lncRNA functions in oligodendrocyte myelination. Genetic ablation of *lncOL1*, a chromatin-associate lncRNA, causes defects in CNS myelination and re-myelination following injury. *LncOL1* interacts with SUZ12, the core component of polycomb repressive complex 2 (PRC2), to promote oligodendrocyte maturation partly *via* transcriptional silencing of gene program that maintains the OL progenitor state (Figure 1I; He et al., 2017).

Notably, not all aforementioned lncRNAs have been exhaustedly examined *in vivo*. Moreover, given lower sequence conservation of lncRNAs than protein-coding genes across species, functional validation using various model organisms, human neural cells and neural organoids are required to assign functions to lncRNAs (Table 1).

## lncRNAs' roles in neurite outgrowth, synaptogenesis, and synaptic plasticity

After neural cells were generated in appropriate numbers, at right times, and in the correct places, they establish functional connections required for normal brain function. To form connections, neurons extend long processes, axons and dendrites, which allow synapse formation (synaptogenesis). Neurite outgrowth and synaptogenesis involve complex regulations on gene expression and signal transduction. Neurons can alter their synaptic connections and the relative strength of individual connections in response to increases or decreases in their activity. This so-called neural plasticity accounts for memory, learning, and cognition, as well as the brain's capability to recover from damage. Compared to studies on lncRNAs' roles in fate specifications of neural cells, little is known regarding lncRNAs' functions in modulating nerite growth, synaptogenesis and neural plasticity, which is partly due to their dynamic features, scarcity of research material and hurdles in functional validation (Puthanveettil et al., 2013).

Nonetheless, emerging evidence indicates both nuclear and synaptic lncRNAs are actively involved in these processes. Comparative sequence analysis of genomic regions covering 150 presynaptic genes discovered highly conserved elements in non-protein coding regions in eight vertebrate species. Many of these “non-exon-associated and non-protein-coding” elements can transcribe and were predicted to form a highly stable stem-loop RNA structure, whereas some conserved noncoding elements correlate with specific gene expression profiles (Hadley et al., 2006). This early work implied that non-coding transcripts are prevalent in genomic regions of presynaptic genes and may have regulatory roles in transcriptional regulation.

### Transcriptional regulation of neurotrophins and synaptic molecules

It has been shown neuronal activity may reshape the epigenetic landscape, thereby dynamically changing transcriptome and neuronal properties (Su et al., 2017). A genome-wide analysis of hyperactive regions of human neocortex removed to treat intractable seizures identified numerous differentially expressed lncRNAs, with a fraction having expression profiles that matched activity-dependent coding genes. Among them, eight lncRNAs were overlapping with or adjacent to differentially expressed protein-coding genes, including reciprocal patterns between *BDNF* (Brain-derived neurotrophic factor) and *BDNF-AS* transcription (Lipovich et al., 2012). BDNF, a member of the neurotrophin family of growth factors, promotes differentiation of new neurons and growth of synapses during development and in adulthood. BDNF signaling is important for long-term memory and its dysfunction have implications in a number of neurodegenerative disorders including Alzheimer's disease (AD). LncRNA *BDNF-AS* negatively regulates BDNF expression by recruiting EZH2, a PRC2 core component, to the *BDNF* locus (Figure 1J; Lipovich et al., 2012). Knockdown of *BDNF-AS* induces BDNF expression in hippocampal neurosphere cultures, which leads to increased neuronal survival and neurite outgrowth (Modarresi et al., 2012). Moreover, *BDNF-AS* transcripts is markedly upregulated in Alzheimer's disease (Shi et al., 2017). In a recent study where differentiated SH-SY5Y neuroblastoma cells were treated with BDNF and subjected to microarray studies, several lncRNAs including *MALAT1*/*NEAT2* and *MIAT*/*GOMAFU* were found to differentially expressed. Interestingly, a few putative microRNA-lncRNA interactions were predicted and seven of the microRNAs are associated with psychiatric and neurodegenerative diseases (Aliperti and Donizetti, 2016).

*Malat1* (Metastasis-associated lung adenocarcinoma transcript 1) is a neuron-enriched nuclear-retained lncRNA associated with nuclear speckle, a structure implicated in pre-mRNA splicing and RNA transport (Figure 1K) (Bernard et al., 2010). Although *Malat1* knockout does not alter gross and histologic morphology of adult mouse brain (Eissmann et al., 2012), *Malat1* depletion in cultured hippocampal neurons resulted in a significant reduction in synaptic density. Conversely, *Malat1* overexpression increased presynaptic bouton density on dendrites. Accordingly, the levels for transcripts of Neuroligin1 (NLGN1) and SynCAM1 post-synaptic proteins were significantly lower in *Malat1*-depleted hippocampal neurons (Bernard et al., 2010). Consistently, *Malat1* maintains survival and neurite outgrowth of Neuro-2a neuroblastoma cells probably via the ERK/MAPK signaling pathway (Chen et al., 2016). The discrepancy between these *in vivo* and *in vitro* results necessitates detail analyses of *Malat1* knockout animals, including structural and functional interrogation of neurites and synapses as well as behavioral studies.

Alterations in expression of genes encoding signaling proteins or ion-channel components can drastically change neuronal excitability. Therefore, regulations of these components can modulate neuronal plasticity. Scaffold protein CASK (Ca2^+^/calmodulin-dependent protein kinase) regulates synapse formation and plasticity during neural development (Martin and Ollo, 1996; Chen and Featherstone, 2011; Slawson et al., 2011). The expression of *Drosophila* CASK is positively regulated by its downstream overlapping lncRNA, *CRG* (CASK regulatory gene), which is neural-specific and is induced during embryonic development. Loss of *CRG* leads to decreased locomotor activity and a defective climbing capability in fly—phenotypes reminiscent of *CASK* mutants and could be rescued by *CASK* overexpression (Li et al., 2012). *KCNA2* encodes a core potassium channel subunit and can be negatively regulated by its antisense RNA *Kcna2-as. Kcna2-as* transcript and KCNA2 proteins are largely reciprocally expressed in DRG neurons. Upon peripheral nerve injuries, *Kcna2-as* expression was triggered by activation of the myeloid zinc finger protein 1 (MZF1) transcription factor. Upregulation of *Kcna2-as* decreased *Kcna2* mRNA, tempered total voltage-gated potassium current and elevated excitability in DRG neurons, ultimately leading to neuropathic pain symptoms. Moreover, blocking the induction of *Kcna2-as* attenuated neuropathic pain following peripheral nerve injury (Zhao et al., 2013).

### Post-transcriptional regulation at synapses

As dendrites and axons usually extend far away from the cell body, local protein translation appears particularly important for maintaining dendritic and axonal function (Job and Eberwine, 2001). Noncoding RNA *BC1*/*BC200* (*BC200* is the primate counterpart of rodent *BC1* RNA) is located in the dendrites and somata of a subset of neurons in the central and peripheral nervous system (Tiedge et al., 1991; Muslimov et al., 1997). The expression of *BC1* RNA in soma and dendrites of hippocampal neurons is induced during synapse formation, and is dependent upon neuronal activity (Muslimov et al., 1998). Accordingly, *BC1* controls protein translation in synaptodendritic microdomains. *BC1* RNA interacts directly with initiation factor eIF4A and poly(A)-binding protein (PABP), preventing association of the 48S pre-initiation complex with mRNA, thus inhibiting the formation of the 48S ribosomal translation initiation complex (Wang et al., 2002). Another report suggested *BC1* RNA binds to the fragile X syndrome protein FMRP to regulate the translation of specific FMRP target mRNAs at synapses (Figure 1L; Zalfa et al., 2003). The brains from *BC1*-null mice have no grossly morphological defects, as were the localization of CaMKIIα and MAP2 dendritic mRNAs (Skryabin et al., 2003). But detailed behavioral studies found *BC1*-deficient mice have defects in exploratory behavior and higher levels of anxiety and increased neuronal excitability, probably due to hyperactive mGluRs (group I metabotropic glutamate receptor)-triggered translation in synapses (Lewejohann et al., 2004; Zhong et al., 2009). These studies implied *BC1/BC200* would contribute to the maintenance of synaptic plasticity. *BC200* levels were greatly reduced in aging brain cortices, but it was significantly up-regulated in AD brains. Relative *BC200* levels correlate with the progression of AD, and its mislocalization (clustered perikaryal localization but not somatodendritic) was observed in advanced AD brains (Mus et al., 2007).

Circular RNAs (circRNAs) have been identified in various species and categorized as a novel type of noncoding RNA (Salzman et al., 2012; Guo et al., 2014). Most circular RNAs arise from “back-splicing,” where a 5′ splice donor joins an upstream 3′ splice acceptor (Jeck and Sharpless, 2014; Xing et al., 2016). Westholm et al. annotated more than 2,500 *Drosophila* circRNAs, many of which contain conserved canonical miRNA seed matches, indicating their impacts on posttranscriptional regulatory networks through acting as molecular sponges for miRNAs. Notably, circRNAs dominantly reside in the *Drosophila* nervous system and their levels increase with age (Westholm et al., 2014). Similarly, thousands of conserved circular RNAs (circRNAs) were found to be highly expressed in mammalian brain. The expression levels of many circRNAs are elevated in neurogenesis and they are more abundantly expressed in synaptic processes than their linear isoforms (Rybak-Wolf et al., 2015; You et al., 2015). Circular RNAs could behave as competing endogenous RNAs (ceRNAs): *ciRS-7* (circular RNA sponge for miR-7), a neuron-enriched circRNA, sequesters miR-7 and prevents miR-7's interactions with target mRNAs (Hansen et al., 2013). These findings point to prospective roles of circRNAs in the brain, especially in synaptogenesis and neural plasticity.

Current knowledge regarding lncRNA's role in synaptogenesis and plasticity is relatively scarce. Studies found lncRNAs seem to regulate circadian or mating behavior in insects and worms (Soshnev et al., 2011; Gummalla et al., 2014), such evidence hasn't been found in mammals yet. Future genome-wide lncRNA knockout studies in mice would unveil the extent and mechanisms how lncRNAs are involved in circuitry dynamics.

## LncRNAs in neurological disorders

Since lncRNAs regulate neural development and function, it's not surprising that mutation or dysregulation of lncRNAs has implications in pathogenesis of mental illness and neurodegenerative diseases such as autism spectrum disorder (ASD), depression, schizophrenia, amyotrophic lateral sclerosis (ALS), Alzheimer's disease and neuropathic pain. Some neural-specific lncRNAs have been emerged as potential therapeutic targets.

LncRNAs related to cognitive functions or synaptic plasticity or other psychiatry diseases, including *GOMAFU, BDNF-AS*, and *DISC2*, may potentially contribute to major depressive disorder (MDD) (Huang et al., 2017). In a microarray-based study, about two thousand lncRNAs were found to be differentially expressed in peripheral blood samples from major depression disorder (MDD) patients (Liu Z. et al., 2014), but their diagnostic and therapeutic implications remain to be elucidated. A recent genome-wide study characterized thousands of lncRNAs to be differentially expressed in ASD peripheral leukocytes. Gene ontology (GO) analysis of these lncRNA gene loci predicted neurological regulations of the synaptic vesicle cycling, long-term depression and potentiation to be mainly involved, including *SHANK2-AS* and *BDNF-AS* (Wang et al., 2015). Similarly, a large-scale study applied RNA sequencing (RNA-seq) of 251 post-mortem samples of frontal and temporal cortex and cerebellum from 48 individuals with ASD and 49 control human subjects, and identified 60 differentially expressed lncRNAs (Parikshak et al., 2016). Twenty of these lncRNAs were previously shown to interact with microRNA (miRNA)–protein complexes, and 9 with the fragile X mental retardation protein (FMRP), whose mRNA targets are enriched in ASD risk genes (Parikshak et al., 2013; Iossifov et al., 2014). These data show that dysregulation of lncRNAs is an integral component of the transcriptomic signature of ASD (Parikshak et al., 2016). LncRNA *GOMAFU*/*MIAT* is downregulated in post-mortem cortical gray matter from schizophrenia (SZ) patients. *GOMAFU* associates with splicing factors SRSF1 (serine/arginine-rich splicing factor 1) and QKI and dysregulation of *GOMAFU* results in aberrant splicing of DISC1 and ERRB4, two SZ-associated genes (Barry et al., 2014). Another study discovered that *GOMAFU* mediates mouse anxiety-like behavior probably via association with BMI1, a key member of the polycomb repressive complex 1 (PRC1), to repress the expression of *beta crystallin* (*Crybb1*), one of the SZ-related genes (Spadaro et al., 2015).

Amyloid precursor protein (APP) is sequentially cleaved by beta-site APP-cleaving enzyme 1 (BACE1), β-secretase, and γ-secretase to generate the toxic Aβ_42_ peptide. Defective Aβ_42_ clearance and elevated BACE1 expression contribute to Aβ_42_ accumulation and AD progression. *BACE1-AS*, the antisense transcript of *BACE1*, can bind to and stabilize *BACE1* transcripts, thus increasing BACE1 protein levels. Interestingly, *BACE1-AS* is induced by Aβ_42_ peptide, leading to increased BACE1 mRNA stability and amyloid accumulation *via* a positive feedback loop. Consistently, expression of BACE1 and *BACE1-AS* is elevated in brains of AD, and knockdown of *BACE1-AS* reduced BACE1 levels *in vivo* (Faghihi et al., 2008; Liu T. et al., 2014). The neuromuscular disorder spinal muscular atrophy (SMA) is caused by insufficient expression of SMN (survival motor neuron) protein, and the primary goal of SMA therapeutics is to increase SMN levels (Lefebvre et al., 1997). LncRNA *SMN-AS1* is enriched in neurons and suppresses SMN expression by recruiting the polycomb repressive complex-2 (PRC2) to *SMN* promoter. Targeting *SMN-AS1* with antisense oligonucleotides (ASOs) increases SMN expression both in cultured neurons and in mice, indicating *SMN-AS1* has potential to be a novel therapeutic target for treating SMA (D'ydewalle et al., 2017).

Large-scale RNA dysregulations are essential molecular hallmarks in neurodegenerative diseases including ALS and FTLD (Frontotemporal lobe dementia; Polymenidou et al., 2012). This is mostly due to the presence of aberrant protein states (proteinopathy) of two essential RNA/DNA binding proteins TDP-43 and FUS (Fused in sarcoma) in affected neurons, including cytosolic translocation, truncation, phosphorylation, ubiquitination, and aggregates formation (Lagier-Tourenne et al., 2010; Da Cruz and Cleveland, 2011). Although TDP-43 and FUS regulate the processing of an array of RNA molecules including non-coding RNAs, no specific RNA was yet identified as major causal factor of ALS and FTLD (Tollervey et al., 2011). The association with TDP-43 or FUS/TLS could allow lncRNAs to carry out their cellular function. On the other hand, the dynamics of association/dissociation of RNAs with TDP-43 or FUS might contribute to TDP-43 and FUS proteinopathies (Yang et al., 2015).

The above findings implicate the correlation between dysregulation of lncRNAs and neurological diseases. However, many were *in vitro* studies with very few mechanistic hints. Thus, we are still far from understanding the extent and mechanisms that lncRNAs are involved in disease brains.

## Advances and challenges of studying lncRNAs *in vivo* and *in vitro*

Although a number of lncRNAs have been found to be involved in most, if not all, aspects of neural circuitry assembly and plasticity, many essential questions remain to be answered. First, in contrast to the abundancy of lncRNAs characterized, very few of them are essential for embryonic development, cell fate choices or circuitry functions. So, as many may ask, are lncRNAs largely transcriptional noise or non-functional? It's quite possible that most lncRNAs only play subtly regulatory roles, and that certain lncRNAs are not normally required but become essential upon neuronal activation or injury. Second, compared to proteins, most lncRNAs have low sequence conservation across species or among homologs, though evolutionary conservation of RNA secondary structures may exist across species. It's, therefore, hard to identify parallel or redundant pathways and related molecular mechanisms. Third, lncRNAs may exert functions *in cis* (transcripts dependent or independent), and/or *in trans* (chromatin remodeling, histone modification, DNA methylation, transcription and splicing regulation etc.). Moreover, the embedding DNA elements that transcribe lncRNAs may have cis-regulatory roles. These conditions greatly confound experiment design and data interpretation for functional studies of lncRNAs. Thus, it's not surprising loss-of-function studies *in vivo* or *in vitro* using different approaches (e.g., RNAi, antisense oligonucleotides, genomic deletion, polyadenylation insertion, promoter deletion/inversion and CRISPR-Cas9 mediated gene inactivation, etc.) may lead to distinct phenotypes (Bernard et al., 2010; Schorderet and Duboule, 2011; Eissmann et al., 2012; Li et al., 2013). Since each technique has advantages and limitations, researchers are required to exhaustedly apply necessary approaches and develop new technologies to elucidate lncRNAs' roles and mechanisms.

Latest breakthroughs in genome engineering technology utilizing CRISPR (clustered regularly interspaced short palindromic repeats) and Cas9 system have dramatically accelerated biomedical researches (Doudna and Charpentier, 2014; Hsu et al., 2014). It has been widely used for generating genetic-modified cells, plants and animals (Cong et al., 2013; Niu et al., 2014; Peng et al., 2014); for disease modeling and genetic corrections (Platt et al., 2014; Cox et al., 2015); as well as for repressing (CRISPRi) or inducing (CRISPRa) gene expressions without altering genomic sequences (Gilbert et al., 2013, 2014; Konermann et al., 2014). In a few proof-of-principle studies, CRISPR-Cas9 has been successfully applied to lncRNA studies in cells and in animals (Ho et al., 2015; Ghosh et al., 2016; Zhu et al., 2016). A genome-scale deletion screening for functional lncRNAs were carried out using a lentiviral paired-guide RNA (gRNA) CRISPR-Cas9 library targeting hundreds of lncRNAs. This screen identified fifty-one lncRNAs that can enhance or slow down human cancer cell growth. Next, nine lncRNA candidates were validated utilizing CRISPR–Cas9-mediated genomic deletion, CRISPRa or CRISPRi, functional rescue and transcriptome profiling. This study indicates high-throughput genome deletion method mediated by CRISPR–Cas9 enables rapid identification of functional non-coding elements (Zhu et al., 2016). Using the minimal CRISPRi (dCas9) system targeting the *roX* locus in the *Drosophila* cells leads to an efficient and specific knockdown of *roX1* and *roX2* lncRNAs. Moreover, this minimal CRISPRi system inhibits *roX* expression efficiently *in vivo* and leads to loss-of-function phenotype, thus validating the method in a multicellular model organism (Ghosh et al., 2016). To explore if certain RNA molecule can exert transactivation or adapter roles, Schechner et al. developed a targeted localization method called CRISPR-Display utilizing Cas9 to deploy RNA cargos to specific DNA loci. A distinct feature of CRISPR-Display is that it makes possible for multiplexing of different functions at multiple loci in the same cell (Shechner et al., 2015). The ever-growing innovation of CRIPSR-Cas9 technique would also enable detection and editing of DNA and RNA with high specificity and sensitivity (Abudayyeh et al., 2016; Gootenberg et al., 2017; Qin et al., 2017). Nonetheless, caution must be taken when applying CRISPR-Cas9 techniques in lncRNA studies: First, deletion of lncRNA genes overlapping protein-coding genes' promoter/enhancer or intron should be avoided; Second, effects of CRISPRa and CRISPRi on promoter/enhancer elements that shared by both lncRNA and protein-coding genes should be taken into account when analyzing phenotypes. Finally, possible off-target effects can be addressed by applying multiple gRNAs and performing rescue experiments.

LncRNAs exert roles by associating with cellular macro-molecules including chromatin, DNA, RNA or proteins. Current biochemical means using RNA-centric or protein-centric strategies can identified these molecules and been extensively reviewed elsewhere (Mchugh et al., 2014). Technology breakthroughs in physics, chemistry, molecular biology and neuroscience would allow researcher to carry on high-throughput investigations of lncRNAs at single-cell, circuitry and animal levels.

## Conclusions and perspectives

One of the primary aim of the BRAIN initiative is to “*identify and provide experimental access to the different brain cell types to determine their roles in health and disease*” (NIH, 2014). However, we are still far from identifying and characterizing the component cells comprising the neural circuits, especially for glial cells. This is partially due to lack of defined biomarkers and dynamic changes of cell properties upon stimulation or depression. Latest advances in cell labeling using genetic and viral means, high throughput purification, e.g., FACS (fluorescence-activated cell sorting) and microfluidic devices, enable researchers to isolate neural cells from embryonic and adult brain under a variety of conditions. Moreover, recent development in profiling transcriptomes and epigenomes from as few as single cells markedly advanced molecular census of neural cell in embryonic and adult brains (Usoskin et al., 2015; Zeisel et al., 2015; Poulin et al., 2016; Tasic et al., 2016; Telley et al., 2016). Tasic et al. established a cellular classification of primary visual cortex in adult mice based on single-cell transcriptome analysis. A total of 49 transcriptomic cortical cell types, including 19 glutamatergic, 23 GABAergic, and 7 non-neuronal types were identified from around 1,600 cells labeled by *Cre* reporters. Interestingly, many transcriptomic cell types showed discrete anatomical and physiological characteristics, thus validating that the single-cell transcriptomic profiles can reflect specific properties of neural cells (Tasic et al., 2016).

Current annotations of brain lncRNAs are unfinished, partly because of the selection of polyadenylated (polyA) transcripts in most studies and RNA-seq libraries not preserving strand information (Miller et al., 2014; Darmanis et al., 2015). Single-cell RNA sequencing is even harder to detect lncRNAs because there's much less starting material and lncRNAs are generally less abundant than protein-coding transcripts. Nonetheless, latest single-cell transcriptome studies indeed correlate lncRNA profiles with developmental stages and cell identities. Using single cell RNA-seq to analyze roughly 100 individual cells from human embryonic stem cells (hESCs) and human preimplantation embryos, Yan *et al*. identified 2,733 previously uncharacterized lncRNAs, many of which are specifically expressed in developmental stages (Yan et al., 2013). In another single-cell study, more than 600 novel multi-exonic lncRNAs were discovered using micro-dissected adult subventricular zone (SVZ) tissues (Luo et al., 2015). Single-cell sequencing of hundreds of human cortical cells revealed that many lncRNAs are enriched in individual cells, and are cortical layer and cell type-specific (Liu et al., 2016), which coincides with previous studies showing lncRNAs provide more cell identity information during the development of mammalian cortex than protein-coding transcripts (Molyneaux et al., 2015). We speculate that lncRNA profiling at sing-cell level, along with high-throughput single molecule fluorescent *in situ* hybridization (smFISH), would greatly advance the census of neurons and glial cell, especially astrocytes, in the context of neural development and plasticity (Femino et al., 1998; Raj et al., 2008). Moreover, transcriptome dissection of cells in cerebral organoid derived from human pluripotent cells or NSPCs would advance our understanding on lncRNAs in brain evolution, development and diseases (Lancaster et al., 2013; Fatehullah et al., 2016).

*N*^6^-Methyladenosine (m^6^A) is a widespread, reversible chemical modification of polyadenylated mRNAs and lncRNAs in eukaryotes, implicated in many aspects of RNA metabolism including regulations of stability, transport and translation (Fu et al., 2014). Antibody-based *N*^6^-methyladenosine (m^6^A) RNA immunoprecipitation followed by high-throughput sequencing (MeRIP-seq) has been developed to profile the transcriptome-wide distribution of m^6^A, revealing m^6^A is distributed in more than 7,000 mRNA and 250 lncRNA transcripts in human cells (Dominissini et al., 2012). In mouse brain, m^6^A is present in mRNA at low levels throughout embryogenesis but increases dramatically by adulthood, suggesting that upregulation of m^6^A levels accompanies neuronal maturation. Moreover, lncRNAs transcribed by RNA polymerase II are also subject to m^6^A methylation, and long intergenic noncoding RNAs (lincRNAs) had significantly higher m^6^A levels than mRNAs or pseudogenes, but its biological implication is largely unknown and awaits future explorations (Meyer et al., 2012; Molinie et al., 2016). Interesting, a long non-coding RNA antisense to *FOXM1* (*FOXM1-AS*) promotes the interaction of m^6^A demethylase ALKBH5 with *FOXM1* nascent transcripts, which facilitates m^6^A demethylation of *FOXM1* pre-mRNA at its 3′UTR. Demethylated *FOXM1* pre-mRNAs have higher affinity with HuR, a RNA binding protein, which stabilizes *FOXM1* to promote glioblastoma stem-like cells self-renewal and tumorigenesis (Zhang et al., 2017).

In summary, the knowledge of lncRNAs in neural circuitry assembly has been greatly expanded in recent years, whereas how lncRNAs exert roles in circuitry function in physiologic and pathologic conditions are much less known. Future studies would use modern genetic labeling, live-imaging, electrophysiology, behavioral and high-throughput means to explore how lncRNA-expressing neural cells are spatiotemporally participated in circuitry assembly and function, which can provide solid evidence that lncRNAs are essential fate and activity markers/determinants of neural cells.

## Author contributions

AW and JW collected references and wrote the review. YL and YZ wrote the review.

### Conflict of interest statement

The authors declare that the research was conducted in the absence of any commercial or financial relationships that could be construed as a potential conflict of interest.
